# Radix Rehmanniae Praeparata Extract Enhances Liver Regeneration Through AMPK-Driven Metabolic Reprogramming

**DOI:** 10.3390/nu17223579

**Published:** 2025-11-15

**Authors:** Changmeng Li, Qi Zheng, Fanghong Li, Yinhao Zhang, Shuwen Duan, Jia Liu, Qi Han, Runping Liu

**Affiliations:** 1 School of Chinese Materia Medica, Beijing University of Chinese Medicine, 11 Bei San Huan Dong Road, Beijing 100029, China; lcm15965726297@163.com (C.L.); bucmszzq@163.com (Q.Z.);; 2 School of Life Sciences, Beijing University of Chinese Medicine, Beijing 100029, China

**Keywords:** Radix Rehmanniae Praeparata, liver regeneration, AMPK, metabolic reprogramming

## Abstract

**Background:** Liver regeneration is essential for restoring hepatic mass after injury or resection, with metabolic reprogramming as a critical driver. Radix Rehmanniae Praeparata (RRP), a traditional Chinese medicine for chronic liver diseases, regulates glucose and lipid metabolism. This study evaluated the effects of RRP on liver regeneration and explored the underlying mechanisms. **Methods:** A 70% partial hepatectomy (PHx) mouse model was employed, and integrated transcriptomic and metabolomic analyses were conducted to characterize the global features of RRP-induced metabolic reprogramming and its association with hepatocyte proliferation. To further validate these findings, the AML12 hepatocyte cell line and primary mouse hepatocytes were used to identify key targets of RRP. **Results:** RRP significantly enhanced liver regeneration, as evidenced by the upregulation of hepatocyte proliferation markers. Transcriptomic, metabolomic, and biochemical analyses showed that RRP promoted lipid catabolism and H3K27ac remodeling-dependent hepatocyte proliferation by increasing acetyl-CoA flux. RRP also enhanced carbohydrate consumption and pentose phosphate pathway, as well as protecting mitochondrial integrity, which contribute to both energy production and nucleotide synthesis during cell cycle progression. Notably, RRP-induced AMPK activation was involved in these metabolic reprogramming events, since pharmacological inhibition of AMPK with Compound C attenuated the promotive effects of RRP on liver regeneration. **Conclusions:** RRP promotes liver regeneration by enhancing metabolic reprogramming mediated by AMPK activation, highlighting its therapeutic potential for metabolic adaptation and postoperative recovery in compromised liver.

## 1. Introduction

The liver possesses a remarkable regenerative capacity to restore functional homeostasis following acute or chronic injury. This regenerative potential primarily relies on the compensatory proliferation of hepatocytes, which rapidly respond to injury signals to replenish lost tissue mass and maintain systemic metabolic functions such as detoxification and nutrient processing [1,2]. Advanced-stage liver diseases, such as decompensated cirrhosis [3], acute-on-chronic liver failure (ACLF) [4], and hepatocellular carcinoma (HCC) [5] are critical global health concerns. However, the insufficient regenerative capacity of livers in these patients is a major cause of liver failure and post-hepatectomy mortality. Therefore, developing novel strategy to enhance liver regeneration has become a key research direction in the fields of regenerative medicine [6].

Current research indicates that metabolic reprogramming, mainly characterized by sharply decreasing blood glucose levels, rapid fat mobilization, and increasing lipid metabolism, plays a critical role in driving liver regeneration [7,8,9]. Our previous study revealed that, during the during the first 12 h post-70% PHx, hepatocytes rapidly reprogrammed their lipid metabolism to ensure robust activation of fatty acid oxidation, concomitant with the suppression of phospholipid and fatty acid ester synthesis [10]. This metabolic shift not only generates large amounts of ATP but also produces abundant acetyl-CoA. The former provides a sufficient energy supply, while the latter facilitates histone acetylation modifications (H3K27ac)-dependent transcriptional rewiring, thereby ensuring hepatocyte proliferation. It is also important to note that these metabolic reprogramming processes depends on the intact function of the mitochondrial network [11,12]. However, observations in acute liver injury models have shown that regenerating hepatocytes frequently exhibit disordered mitochondrial cristae structures and reduced membrane potential, indicative of a compensatory imbalance [13,14]. This overactive metabolic state of mitochondria can lead to a burst of reactive oxygen species (ROS), which can disrupt mitochondrial membrane integrity and trigger the release of pro-apoptotic factors, such as cytochrome c [15]. These factors activate caspases and initiate the apoptotic cascade, with the leakage of pro-apoptotic signals forming a critical bottleneck that constrains liver regenerative efficiency [16]. Targeting the delicate balance between metabolic reprogramming and mitochondrial network function may represent a novel strategy to promote liver regeneration.

Radix Rehmanniae Praeparata (RRP), a classic Traditional Chinese Medicine (TCM) herb derived from the processed root of Rehmannia glutinosa Libosch (Scrophulariaceae), is traditionally revered for its actions to tonify the liver and kidneys, replenish vital essence, and nourish blood and “yin” [17,18]. From the perspective of metabolism reprogramming, Wang et al. found that RRP aqueous extract can effectively improve mitochondrial respiratory function and address energy metabolism issues in the prefrontal cortex of rats with attention deficit hyperactivity disorder [19]. Specifically, RRP contains key bioactive components, such as catalpol, verbascoside, and pinoresinol, which belong to the iridoid glycoside family which have been widely reported to improve glucose and lipid metabolism through antioxidant activity, mitochondrial function enhancement, cellular aging suppression, and energy metabolism regulation [20,21]. These indirect pieces of evidence suggest that RRP may potentially participated in the process of metabolic reprogramming, thereby influencing the process of liver regeneration.

In the current study, we use a 70% PHx mouse model to evaluate the promotive effects of the RRP aqueous extract (referred as RRP) on liver regeneration and employ integrated transcriptomic-metabolomic analysis and multimodal validation approaches to elucidate the underlying mechanisms. Informed by multi-omics evidence, we investigate the effects of RRP on AMPK, a shared regulator of carbohydrate, ribonucleic acid, and lipid metabolism, in AML12 hepatocytes. Additionally, we characterized the potential modulatory effects of RRP on mitochondrial function in primary hepatocytes. Although our previous studies have emphasized the importance of metabolic reprogramming in the initiation of liver regeneration [22], whether it is targetable remains unclear. Our findings may support the use of RRP-based therapeutic strategies as perioperative intervention to enhance liver regeneration.

## 2. Method

### 2.1. The Preparation of RRP Aqueous Extract

RRP was bought from Beijing Tongrentang (Group) Co., Ltd. in Beijing, China, (batch number No. 23101301). The chemical composition and major constituents of the RRP aqueous extract, including catalpol, verbascoside (acteoside), rehmannioside D, and 5-hydroxymethylfurfural, were confirmed and quantified in our previous study using HPLC and LC-MS/MS to ensure the consistency and quality of the extract [22]. An amount of 30 g of the raw material was ground and subjected to aqueous extraction by immersion in 240 mL of distilled water for 60 min. The mixture was then heated and boiled for extraction, done twice at boiling temperature for 60 min each time. After collecting the extracted solutions, they were condensed using rotary evaporation under reduced pressure. The solution was then filtered 2 times to remove any particles. Finally, the filtered liquid was dried using vacuum freeze-drying to make it lyophilized powder, which was kept at −20 °C.

### 2.2. Animal Studies

A mouse model in which 70% partial hepatectomy (PHx) was performed on 7-week-old male C57BL/6J mice was used to construct a liver regeneration model. Under isoflurane anesthesia (4% induction, 2.5% maintenance), a midline abdominal incision was made below the xiphoid to ligate and excise the median and left lateral liver lobes with strict anesthesia and surgical time limits (15 min per mouse). Sham operated mice were accepted to the same procedure. Post-surgery, the abdomen was sutured and disinfected, and mice were monitored during recovery [10]. All mice were sacrificed after surgery at a specific time to collect their serum and liver samples for further experiments. All animal procedures were approved by the Institutional Animal Care and Use Committee of Beijing University of Chinese Medicine and strictly adhered to institutional and national ethical guidelines. Ethical approval for all animal procedures was obtained from the Ethical Review Committee of Animal Experiments in Beijing University of Chinese Medicine (BUCM20250610-001, approval data 17 September 2020).

Based on the 2020 Chinese Pharmacopoeia and some studies, the in vivo dosing regimen for RRP aqueous extract was established. In the dose optimization study, the mice were divided into five groups (*n* = 6/group): (1) Sham group, (2) 48 h post-partial hepatectomy (PHx), (3) 48 h post-PHx + RRP_L (2.5 g/kg), (4) 48 h post-PHx + RRP_M (5 g/kg), and (5) 48 h post-PHx + RRP_H (10 g/kg). After one week of acclimatization, PHx was performed on surgical groups. RRP or 0.5% saline (control) was administered via oral gavage 3 days pre-surgery (4 h before surgery) and daily postoperatively. For the subsequent formal experiment, the optimal dose (5 g/kg) was selected. Mice (7-week-old, n = 9/group) were allocated into: (1) Sham group, (2) 24 h post-PHx, (3) 24 h post-PHx + RRP, (4) 96 h post-PHx, and (5) 96 h post-PHx + RRP, following identical acclimatization and dosing protocols.

### 2.3. The Assessment of Liver Coefficient and Hepatic Enzyme Levels

The liver coefficient was calculated by dividing the liver weight by the body weight, then multiplying by 100 to express it as a percentage. This ratio is used to assess liver size relative to body size in both healthy and diseased states. Assay kits (Jiancheng, Nanjing, China) were used to measure the activity of serum aspartate aminotransferase (AST) and alanine aminotransferase (ALT).

### 2.4. Acetyl-CoA Assay

Acetyl-CoA levels in snap-frozen liver tissues were quantified by Solarbio assay kit. Tissues were homogenized in ice cold lysis buffer and centrifuged, and supernatant was assayed in duplicate. OD340 nm was measured at 20 s and 80 s after reagent addition. Acetyl-CoA content was calculated as ΔOD (80 s–20 s)/sample weight, depending on manufacturer’s protocol [23]. The acetyl-CoA levels in the regenerating liver were measured in Sham group, 24 h post-PHx group, and 24 h post-PHx+RRP group.

### 2.5. Hematoxylin and Eosin (H & E) Staining

Mouse liver tissues were fixed in 4% paraformaldehyde for 7 days, paraffin-embedded, and sectioned into 5 μm slices. Hematoxylin and eosin (H&E) staining was performed according to established protocols [24]. Histological images were acquired and analyzed by using the Aperio Versa whole slide scanning system (Leica Microsystems, Wetzlar, Germany).

### 2.6. Immunohistochemistry Analysis and Immunofluorescence

Paraffin sections underwent antigen retrieval; endogenous peroxidase blocking with 3% H_2_O_2_; and overnight incubation with primary antibodies against Cyclin D1 (Ccnd1) (Proteintech, 1:100), Marker of proliferation Ki-67 (Ki67) (Proteintech, 1:600), and H3K27ac (CST, 1:100) in a wet chamber at 4 °C. Next, the slices were washed and incubated with goat anti-mouse/rabbit IgG HRP polymer secondary antibody (ZSGB-BIO, Beijing, China) [25]. The goat anti-mouse/rabbit IgG HRP polymer secondary antibody was used as a ready-to-use reagent according to the manufacturer’s instructions. Images were acquired using an Aperio Versa scanner (Leica). AOD denotes Average Optical Densit, a quantitative index of histochemical (IHC) staining intensity. AOD = integrated optical density (IOD)/stained area on DAB-channel images after color deconvolution and background correction.

For tissue immunofluorescence, deparaffinized and rehydrated sections went through antigen retrieval, followed by blocking to reduce nonspecific binding. Samples were incubated overnight at 4 °C with primary antibody TOM20 (Proteintech, 1:500). After washing with PBS, slices were incubated with corresponding fluorescent secondary antibody (Alexa Fluor 594-conjugated anti-rabbit IgG, CST, #8889S, with a 1:1000 dilution) for 1 h at room temperature, counterstained with DAPI, and imaged using an Olympus FV3000 confocal microscope (Tokyo, Japan).

### 2.7. AML12 Cell Culture and Treatment

AML12 cells were first placed in dishes and left overnight to stick to the surface. After this, the medium was changed to low-serum DMEM high-glucose medium (1% fetal bovine serum and 1% penicillin-streptomycin) for 1 h to prepare the cells.

In normal conditions, cells were treated with RRP aqueous extract. The extract was mixed into complete medium at three doses: low (0.05 mg/mL), medium (0.1 mg/mL), and high (0.15 mg/mL). Cells were exposed to these doses for 24 h. For high fat experiments, a special medium called OAPA was used. OAPA was made by mixing oleic acid (OA) and palmitic acid (PA) in a 2:1 ratio. The stock solution had 50 mM OA and 25 mM PA. This stock was diluted 100 times in complete medium to get the final OAPA medium (500 μM OA and 250 μM PA). Cells were first switched to this OAPA medium for 1 h. After that, RRP was added at the same three doses (0.05, 0.1, 0.15 mg/mL) for 24 h. Each experiment was repeated three times. After treatment, cells were collected at planned times. Proteins were extracted and tested by Western blot to study metabolic changes.

### 2.8. Mouse Liver Primary Cell Culture and Treatment

Initially, 7-week-old male C57BL/6J mice were divided into groups, with the experimental group receiving oral RRP administration for three consecutive days while the control group was given saline. Before surgery, solutions were prepared. The EGTA perfusion buffer contained the following final concentrations: NaCl (8000 mg/L), KCl (400 mg/L), NaH_2_PO_4_ (76.63 mg/L), Na_2_HPO_4_ (120.45 mg/L), HEPES (2380 mg/L), NaHCO_3_ (350 mg/L), EGTA (190 mg/L), and glucose (900 mg/L). Williams’ E medium was similarly prepared and supplemented with dexamethasone and L-thyroxine stock solutions. Collagenase IV-containing perfusion and digestion buffers were warmed to 39 °C before use. Surgical instruments were sterilized by autoclaving, and the workstation is UV-treated. Mice were anesthetized using isoflurane inhalation, maintained at 3% concentration, and positioned supine on the surgical platform. After abdominal disinfection, the peritoneal cavity was opened to expose the liver vasculature. The inferior vena cava was cannulated for EGTA buffer perfusion at 4 mL/min, followed by increased flow after portal vein transection. Once the liver becomes pale, it was excised, minced in digestion buffer, and enzymatically dissociated at 37 °C with gentle agitation [26]. Throughout the procedure, strict aseptic techniques and temperature control were maintained to ensure hepatocyte viability.

### 2.9. Mitochondrial Function and Cellular Metabolic Status of Liver Cells via Seahorse XFE24

Primary mouse hepatocytes were isolated through portal vein perfusion with collagenase IV-containing buffer (7 mL/min, 37 °C), followed by tissue digestion, filtration (100 μm strainer), and centrifugation (60× *g*, 4 °C, 5 min ×2) to purify cells, which were resuspended in Williams’ E complete medium. For Seahorse XFe24 analysis, cells were seeded at 10,000 cells/well in assay medium (1 mM pyruvate, 2 mM glutamine, 10 mM glucose), pre-incubated at 37 °C (CO_2_-free) for 1 h, and subjected to a mitochondrial stress test. Sensor cartridges were hydrated overnight with Seahorse XF calibrant, and compounds (oligomycin: 2 μM; FCCP: 1.5 μM; rotenone/antimycin A: 0.5 μM each) were injected sequentially to measure oxygen consumption rate (OCR) and extracellular acidification rate (ECAR). Key parameters included basal respiration, ATP production, maximal respiratory capacity, and glycolytic flux. Under normal conditions, RRP aqueous extract enhanced mitochondrial efficiency and glycolytic capacity. In high-fat models (OAPA: 500 μM oleic/palmitic acid), RRP restored mitochondrial function by increasing spare respiratory capacity and reducing oxidative stress markers. Data were normalized to cell counts and analyzed via Wave software (Version 2.6, Agilent Technologies, Santa Clara, CA, USA) and GraphPad Prism (Version 9.1.1), with triplicate technical and biological replicates ensuring robustness [27].

### 2.10. Western Blotting Analysis

Proteins were extracted from cells and liver tissues using RIPA lysis buffer (BIORIGIN), resolved via SDS-PAGE, and transferred electrophoretically to PVDF membranes (Merck Millipore, Darmstadt, Germany). Membranes were probed overnight at 4 °C with primary antibodies.

AMPK (ABACM, 1:1000), phosphorylated AMP-activated protein kinase (p-AMPK) (ABACM, 1:1000), Acetyl-CoA carboxylase (ACC) (proteintech, 1:10,000), phosphorylated Acetyl-CoA carboxylase (p-ACC) (proteintech, 1:1000), followed by one hour incubation with species matched secondary antibodies (Proteintech, HPR-conjugated Goat Anti-Rabbit lgG(H+L), 1:20,000; Proteintech, HRP-conjugated Affinipure Goat Anti-Mouse IgG(H+L) 1:20,000). Protein bands were detected using a ChemiDoc imaging system (Bio-Rad, Hercules, CA, USA) [28].

### 2.11. RNA-Sequencing Analysis and Quantitative Real-Time PCR (qPCR)

Total RNA was isolated from mouse liver tissues using Trizol reagent, with concentration and purity assessed via NanoPhotometer^®^ spectrophotometer (IMPLEN, Beijing, China). After mRNA purification, cDNA fragments were selected for library preparation and sequenced on the Illumina NovaSeq platform using standardized protocols. Normalized gene expression data were analyzed with the edge R package to identify differentially expressed genes (DEGs). Functional enrichment of GO terms and KEGG pathways was conducted via the clusterProfiler R package, supplemented by GSEA and heatmap visualization for comprehensive DEG interpretation. For qPCR, cDNA was synthesized from total RNA using HiScript III RT SuperMix (Vazyme Biotech, Nanjing, China, R323-01), and target gene expression levels were quantified with Hprt1 as normalized control [10]. The primer sequences used were as follows: Hprt1 forward: CCGAGGATTTGGAAAAAGTGTT, reverse: CATCTCCTTCATGACATCTCGA; CCND1 forward: CGTATCTTACTTCAAGTGCGTG, reverse: ATGGTCTCCTTCATCTTAGAGG; KI67 forward: CCTGTCACTCCAGATCAGAACT, reverse: GGAGGCAGTCTTCATAGTCTTCT.

### 2.12. Untargeted Metabolomics

Liver tissues (~100 mg) were homogenized in liquid nitrogen, extracted with 80% methanol/0.1% formic acid, and centrifuged (15,000× *g*, 4 °C, 20 min). Supernatants were diluted to 53% methanol, re-centrifuged, and analyzed via LC-MS/MS (Vanquish UHPLC-Orbitrap Q Exactive HF system) using a Hypesil GOLD column (100 × 2.1 mm, 1.9 μm) with a 12 min gradient (0.2 mL/min). MS parameters included 3.5 kV spray voltage, 320 °C capillary, and polarity-specific eluents. Data were processed in Compound Discoverer 3.1 (alignment: 0.2 min RT tolerance, 5 ppm mass error) and matched against mzCloud/KEGG/HMDB/LIPID Maps. Metabolites with VIP > 1, *p* < 0.05, and |FC| ≥ 2 were deemed differential. Enriched pathways (KEGG, *p* < 0.05) were identified. Data are deposited in OMIX (OMIX005833/OMIX005834).

### 2.13. Combined Analysis of Transcriptomics and Metabolomics Isolation

To perform transcriptome-metabolome correlation analysis in liver regeneration studies, begin by extracting expression levels (FPKM) of cell cycle/proliferation-related genes from transcriptomics data and metabolite abundance data from metabolomics. Use statistical tools like the R package psych to calculate Pearson’s correlation coefficients (r) and *p*-values between gene expression and metabolite abundance, generating a correlation matrix to identify significant metabolite-gene pairs. For visualization, plot clustered heatmaps to highlight patterns of co-varying metabolites and genes. Calculate coefficients (R^2^, slope) and visualize trends with scatterplots, fitted lines, and 95% confidence bands. This approach links metabolic reprogramming to transcriptional regulation during biological processes such as liver regeneration.

### 2.14. Statistical Analysis

The results are shown as average values ± standard error (S.E.M.), calculated from at least three separate experiments. Data analysis was done using GraphPad Prism 8 software (GraphPad, Inc., San Diego, CA, USA). We used one-way ANOVA for statistical testing, followed by Tukey’s HSD test to compare differences between groups. *p*-values less than 0.05 were taken as significant.

## 3. Results

### 3.1. RRP Promotes Liver Regeneration in PHx Mice

**Figure 1:**
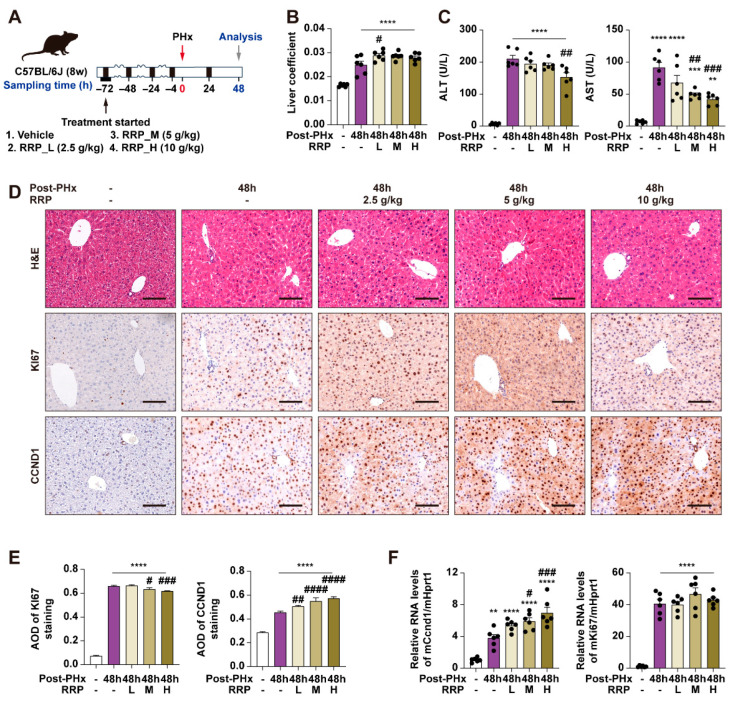
Different doses of RRP promote liver regeneration at 48 h post-PHx in mice. (**A**) Experimental design of PHx surgery, RRP administration (L, M, and H correspond to 2.5, 5, or 10 g/kg, respectively), and sample collection at 48 h post-PHx. (**B**) Liver coefficient. (**C**) Serum levels of ALT and AST. (**D**) H & E staining and immunohistochemistry of Ki67 and CCND1 in liver sections at 48 h post-PHx in the control and RRP-treated groups (2.5, 5, and 10 g/kg). Scale bar = 100 μm. (**E**) AOD quantification of KI67 and CCND1. (**F**) Relative mRNA expression of Ccnd1 and Ki67 in liver from each group was determined by qPCR and normalized using Hprt1 as an internal control. Statistical significance: ** *p* < 0.01, *** *p* < 0.001, **** *p* < 0.0001 as compared with the control group; ^#^ *p* < 0.05, ^##^ *p* < 0.01, ^###^ *p* < 0.001, ^####^ *p* < 0.0001 as compared with the PHx group (*n* = 6 for mice).

**Figure 2:**
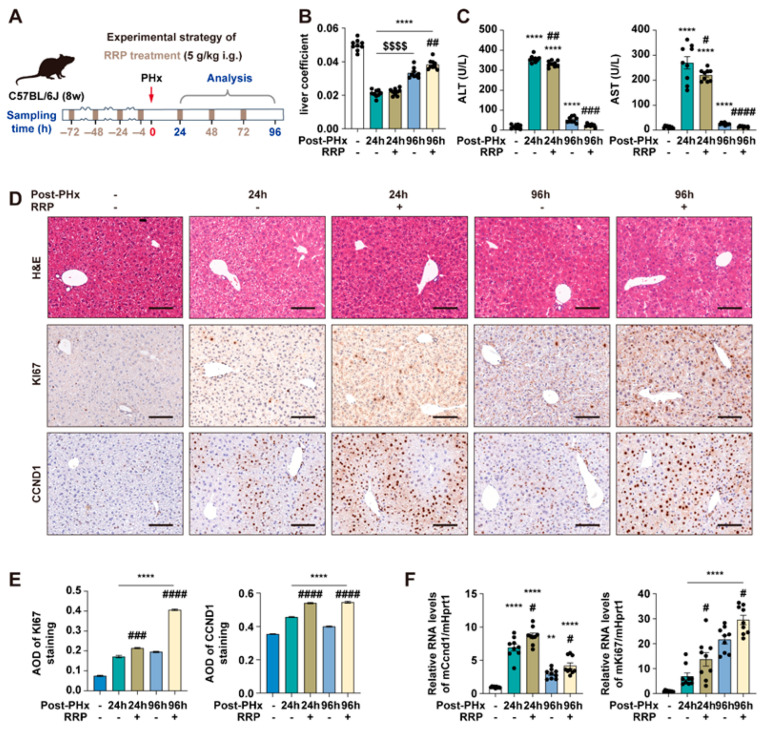
RRP promotes early and sustained hepatocyte proliferation and alleviates liver injury after PHx. (**A**) Experimental timeline of 70% PHx and RRP treatment (5 g/kg). (**B**) Liver coefficient. (**C**) Serum levels of ALT and AST. (**D**) H & E, KI67, and CCND1 staining of liver sections at 24 h and 96 h post-PHx with or without RRP treatment. Scale bar = 100 μm. (**E**) AOD quantification of KI67 and CCND1. (**F**) Relative mRNA expression of Ccnd1 and Ki67 in liver, normalized to Hprt1. Statistical significance: ** *p* < 0.01, **** *p* < 0.0001 as compared with the control group; ^#^ *p* < 0.05, ^##^ *p* < 0.01, ^###^ *p* < 0.001, ^####^ *p* < 0.0001 as compared with the PHx group without RRP treatment; ^$$$$^ *p* < 0.0001 between indicated groups (*n* = 6 for mice).

At 48 h post-PHx, within the acceleration phase of liver regeneration, RRP slightly increased the liver coefficient (Figure 1A,B), while both serum ALT and AST levels decreased with increasing RRP doses, indicating reduced liver injury (Figure 1C). Immunohistochemical analysis also showed enhanced hepatocyte proliferation after RRP treatment, as evidenced by increased Ki67 and Ccnd1 expression (Figure 1D–F). Among the tested doses, 5 g/kg provided the most consistent and optimal balance of efficacy (increased proliferation markers, decreased injury indicators) without signs of toxicity or excessive response, and also falls within the clinically equivalent range based on Chinese Pharmacopoeia. Therefore, 5 g/kg was selected for subsequent experiments (Figure 2A). The temporal effects of RRP were then evaluated at 24 h (initiation phase) and 96 h (acceleration phase) post-PHx using the 5 g/kg dose. RRP consistently increased liver coefficients at both time points (Figure 2B), with reduced serum ALT and AST levels (Figure 2C). Immunohistochemistry showed a few Ki67-positive hepatocytes in untreated group at 24 post-PHx, mainly scattered in the central lobule and RRP increased their numbers at both time points (Figure 2D,E). The q-PCR analysis further confirmed RRP-induced Ki67 and Ccnd1 expression at both 24 h and 96 h (Figure 2F). Collectively, these results indicated that RRP promoted both early and sustained hepatocyte proliferation and alleviated liver injury during liver regeneration.

### 3.2. RRP Promotes Cell Cycle Progression and Metabolic Reprogramming During Early Liver Regeneration

**Figure 3:**
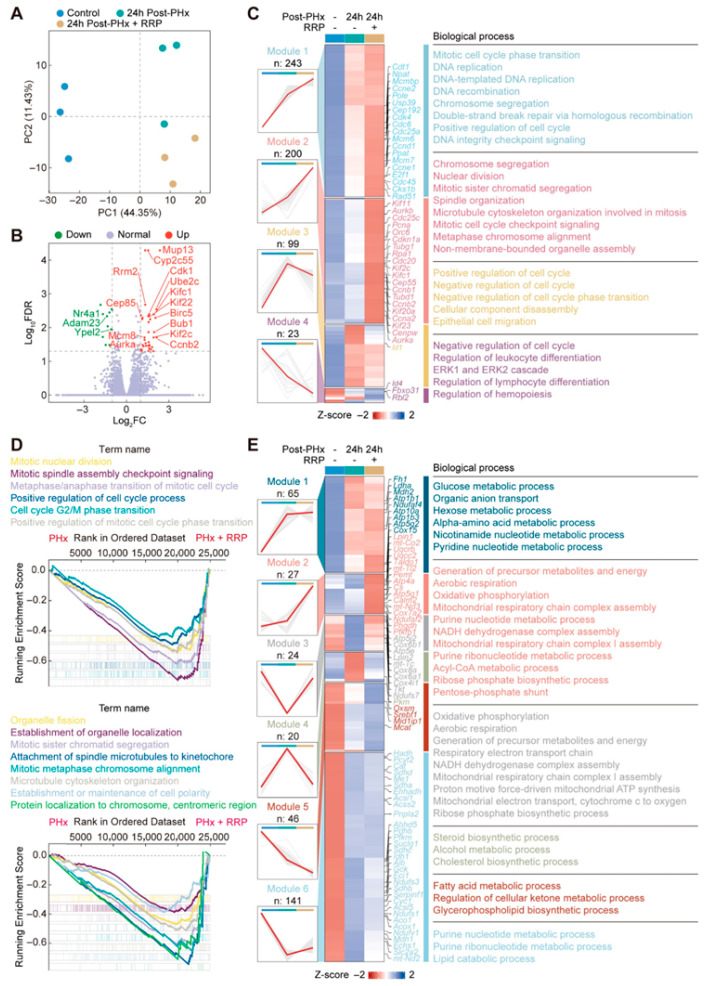
RRP promotes cell cycle progression and induces transcriptional metabolic reprogramming during early liver regeneration. (**A**) Principal component analysis of transcriptomic profiles. (**B**) Volcano plot of differentially expressed genes. (**C**) Cluster heatmap of genes enriched in cell division-related GO terms. (**D**) GSEA analysis plot showing enrichment of cell cycle-related gene sets. (**E**) Cluster heatmap of genes involved in cellular metabolic processes.

24 h post-PHx was chosen for multi-omics analysis as it marks the initiation phase of liver regeneration, when key transcriptional and metabolic programs were activated. To explore how RRP influenced the liver regeneration, we performed RNA-seq on liver tissues from control, 24 h post-PHx, and 24 h post-PHx + RRP mice. Principal component analysis (PCA) revealed distinct transcriptional profiles among the three groups, with PC1 and PC2 accounting for 44.35% and 11.43% of the total variance, respectively (Figure 3A). Compared with the 24 h post-PHx group, RRP treatment induced further transcriptional remodeling, as reflected by distinct gene expression changes in key regulators of the cell cycle, mitosis, and metabolic adaptation (Figure 3B). Heatmap analysis (Figure 3C) showed that RRP enhanced the expression of genes involved in cell cycle progression and mitosis, including members of the MCM family, cyclins, and CDKs. Genes associated with DNA repair, such as RAD51 Recombinase (RAD51) and DNA primase subunit 1 (Prim1); spindle assembly, including Aurora kinase A (Aurka) and Aurora kinase B (Aurkb); and chromosome segregation, such as centromere protein W (Cenpw), were significantly upregulated. Meanwhile, negative regulators of the cell cycle, including retinoblastoma-like protein 2 (Rbl2), inhibitor of DNA binding 1 (Id1), and cyclin-dependent kinase inhibitor 1A (Cdkn1a), exhibited reduced expression. Consistently, GO biological process and GSEA enrichment analyzed confirmed that RRP activated pathways related to DNA replication, repair, chromosome segregation, and mitosis, thereby promoting more efficient hepatocyte proliferation during regeneration (Figure 3D).

To investigate changes in energy metabolism, we analyzed 323 genes associated with mitochondrial function, which were grouped into six distinct clusters based on their expression patterns (Figure 3E). RRP significantly downregulated genes involved in lipid biosynthesis, such as Srebf1 and Mcat, suggesting a metabolic shift away from lipogenesis toward more energy efficient pathways. This supported the hypothesis that RRP suppresses anabolic lipid pathways to prioritize energy production, thereby facilitating regenerative processes. In parallel, RRP treatment enhanced the expression of genes associated with oxidative phosphorylation, including mitochondrially encoded NADH dehydrogenase 3 (mt-Nd3), NADH:ubiquinone oxidoreductase core subunit S7 (Ndufs7), cytochrome c oxidase subunit 7A2 (Cox7a2), and ATP synthase F1 complex epsilon subunit (Atp5e), indicating improved mitochondrial respiratory capacity and elevated ATP production to meet the high energy demands of hepatocyte proliferation. This metabolic adjustment aligned with our proposed model of energy reallocation during early liver regeneration. Notably, a novel finding in this study was the upregulation of genes involved in the pentose phosphate pathway, including transketolase (Tkt) and transaldolase 1 (Taldo1), a central branch of glucose metabolism that generates ribose-5-phosphate for nucleotide synthesis and NADPH for biosynthetic and antioxidant functions. This was accompanied by the increased expression of genes involved in nucleotide biosynthesis, suggesting a coordinated upregulation of metabolic programs that supply both building blocks for DNA replication and redox balance for proliferating cells. As anticipated, these results supported our initial hypothesis that RRP promoted liver regeneration not only by enhancing cell cycle progression and DNA repair, but also by orchestrating metabolic reprogramming across multiple pathways, including lipid catabolism, mitochondrial respiration, and nucleotide-generating glucose metabolism.

### 3.3. RRP Reshapes Lipid Metabolism to Promote Cell Cycle Advancement in Liver Regeneration

**Figure 4:**
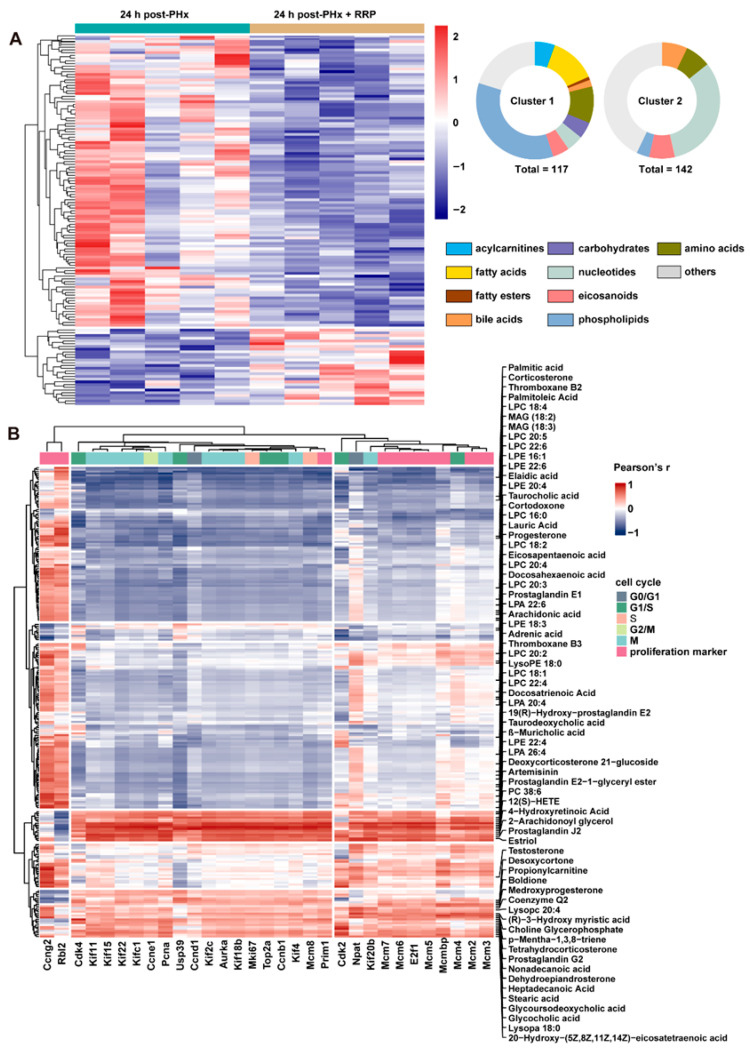
RRP remodels lipid metabolism and links lipid-derived metabolites to hepatocyte cell cycle activation. (**A**) Metabolite heatmap with abundance clustering and compound classification. (**B**) Heatmap of correlations between metabolites and cell cycle genes.

The metabolic rewiring predicted by transcriptomic analysis was further supported by untargeted metabolomics profiling (Figure 4A). A total of 259 significantly altered metabolites were identified and grouped into two major clusters through hierarchical clustering. Cluster 1, containing 117 metabolites, was characterized by consistent downregulation following RRP treatment. Within this cluster, phospholipids accounted for over 40%, followed by fatty acids (approximately 20%) and amino acids (over 10%). In contrast, Cluster 2 consisted of 142 upregulated metabolites, with nucleotide-related compounds representing the majority. At 24 h post-PHx, RRP treatment led to a further reduction in free fatty acids and phospholipids, accompanied by increased levels of bile acids and steroid derivatives. This pattern indicates that RRP actively participates in early-stage lipid metabolic reprogramming during liver regeneration. To further examine the functional relevance of these alterations, we investigated the association between differentially abundant lipid metabolites and genes involved in hepatocyte proliferation (Figure 4B). The correlation analysis revealed strong negative associations between several phospholipids and critical regulators of cell cycle progression, particularly genes related to mitosis, including cyclin B1 (Ccnb1), centromere protein F (Cenpf), BUB1 mitotic checkpoint serine/threonine kinase (Bub1), and aurora kinase B (Aurkb). These phospholipids, phosphatidylcholine (LPC) 22:6, phosphatidylethanolamine (PE) 38:6, and phosphatidylinositol (PI) 40:8, commonly featured polyunsaturated fatty acid chains., commonly featured polyunsaturated fatty acid chains, suggesting that the accumulation of PUFA-rich phospholipids may suppress hepatocyte proliferation. Similarly, several free fatty acids such as palmitic acid, arachidonic acid, and oleic acid were negatively correlated with proliferation-associated genes, consistent with prior studies indicating that excessive levels of saturated or unsaturated fatty acids can induce cellular stress or promote differentiation.

In contrast, a subset of lipids showed positive correlations with genes driving cell cycle progression. Prostaglandin derivatives such as Prostaglandin J2 and Prostaglandin E2 were elevated in the RRP-treated group and were positively associated with genes regulating DNA synthesis and mitotic entry, potentially through signaling mediated by E-type Prostaglandin Receptor. Steroid hormones including progesterone and testosterone were also positively correlated with genes related to chromosomal stability and DNA replication, such as centromere protein F (Cenpf) and minichromosome maintenance complex component 3 (Mcm3), suggesting a role for nuclear hormone receptor pathways in supporting regeneration. In addition, increased levels of taurocholic acid were positively associated with Cdk2 and cyclin E1 (Ccne1), implicating the importance of farnesoid X receptor (FXR)-mediated signaling in the promotion of G1/S phase transition. Collectively, these findings indicate that the lipid metabolic remodeling induced by RRP is closely linked to the regulation of hepatocyte proliferation.

### 3.4. RRP-Induced AMPK Activation Is Involved in Lipid Reprogramming During Liver Regeneration

**Figure 5:**
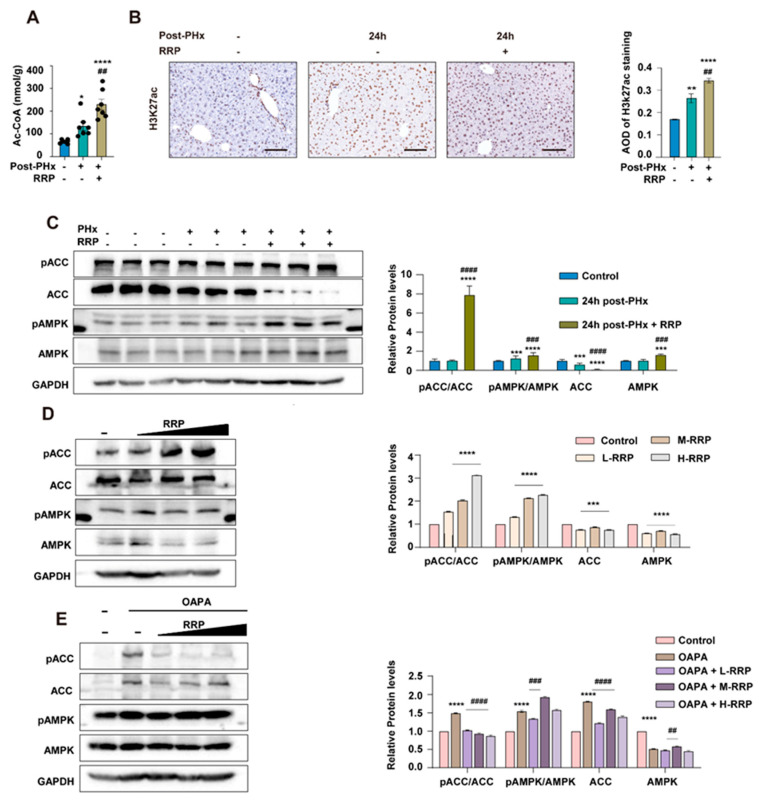
RRP activates AMPK signaling and increases acetyl-CoA production to support epigenetic remodeling. (**A**) Quantification of hepatic acetyl-CoA levels at 24 h post-PHx with or without RRP. (**B**) Immunohistochemical detection of H3K27ac and its quantification. (**C**) Western blot analysis of pACC, ACC, pAMPK, and AMPK levels in liver tissues after PHx and RRP treatment. (**D**) Regulation of AMPK signaling proteins by different concentrations of RRP in vitro. (**E**) Effects of RRP on AMPK pathway activation in hepatocytes under OAPA-induced lipid overload. Scale bar = 100 μm. Statistical significance: * *p* < 0.05, ** *p* < 0.01, *** *p* < 0.001, **** *p* < 0.0001 as compared with the control group; ^##^ *p* < 0.01, ^###^ *p* < 0.001, ^####^ *p* < 0.0001 as compared with the model group (*n* = 6 for mice and *n* = 3 for cell experiments).

The integrative analysis of transcriptomic and metabolomic data revealed that RRP regulated lipid metabolism in a manner highly consistent with the endogenous lipid metabolic reprogramming that naturally occurred during liver regeneration. Our previous studies revealed that such reprogramming, featuring enhanced FAO and reduced phospholipid synthesis, contributed to hepatocyte cycle entry by supplying acetyl-CoA and remodeling the H3K27ac epigenetic landscape. In support of this mechanism, we found that RRP treatment significantly elevated hepatic acetyl-CoA levels 24 h after PHx (Figure 5A). Immunohistochemical staining further showed an increased number of H3K27ac-positive hepatocytes (Figure 5B), confirming that RRP promoted metabolic and associated epigenetic remodeling. AMPK signaling is crucial in regulating the balance between fatty acid oxidation (FAO) and fatty acid (FA) synthesis, as well as the acetyl-CoA flux. In vivo, RRP administration increased pAMPK and its downstream target ACC (pACC), accompanied by reduced total ACC protein levels (Figure 5C). This reduction in total ACC suppressed fatty acid synthesis and preserved acetyl-CoA availability for energy production and histone acetylation, thereby supporting accelerated liver regeneration. Consistent findings were observed in AML12 hepatocytes, where RRP enhanced AMPK activation under both basal conditions and OAPA-induced lipid metabolic stress. Increased pAMPK and pACC levels, along with decreased total AMPK and ACC protein, confirmed the regulative effects of RRP on lipid metabolism (Figure 5D,E)

**Figure 6:**
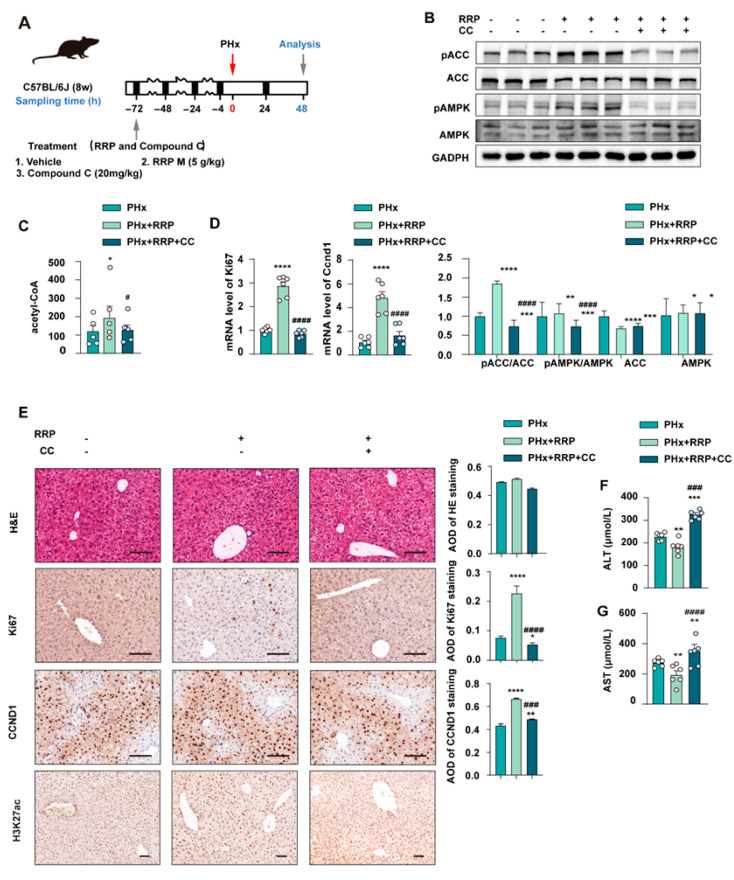
AMPK inhibition impairs RRP-induced metabolic remodeling and hepatocyte proliferation post-PHx. (**A**) Experimental timeline showing PHx, RRP administration (5 mg/kg), and Compound C (20 mg/kg) intervention. (**B**) Western blot analysis of pACC, ACC, pAMPK, and AMPK protein levels. (**C**) Hepatic acetyl-CoA levels. (**D**) Relative mRNA expression of Ccnd1 and Ki67 in liver from each group was determined by qPCR and normalized using Hprt1 as an internal control. (**E**) H&E and immunohistochemical staining for KI67, CCND1, and H3K27ac, with AOD quantitative analysis. (**F**,**G**) Serum levels of ALT and AST. Scale bar = 100 μm. Statistical significance: * *p* < 0.05, ** *p* < 0.01, *** *p* < 0.001, **** *p* < 0.0001 as compared with the PHx group; ^#^ *p* < 0.05, ^###^ *p* < 0.001, ^####^ *p* < 0.0001 as compared with the PHx + RRP group (*n* = 6 for mice).

To further verify the role of AMPK in RRP-mediated liver regeneration, Compound C (CC), a selective AMPK inhibitor, was co-administered with RRP in the PHx model (Figure 6A). As depicted in Figure 6B, CC blocked RRP-induced activation of AMPK signaling, as shown by reduced phosphorylation of AMPK and ACC, and inhibited RRP-related production of intracellular acetyl-CoA production (Figure 6C). Compared with the RRP group, the RRP+CC group showed significantly lower mRNA levels of Ccnd1 and Ki67 (Figure 6B) and fewer Ccnd1 and Ki67 positive hepatocytes (Figure 6D,E), indicating impaired regeneration. Serum ALT and AST levels were higher in the RRP+CC group than in the RRP group (Figure 6F,G), suggesting increased liver injury. Together, these findings underscore the pivotal role of AMPK in mediating the pro-regenerative effects of RRP by orchestrating both metabolic reprogramming and cell cycle advancement. Inhibition of AMPK disrupted this coordination, leading to impaired metabolic adaptation and compromised liver regeneration.

### 3.5. RRP Promotes Glucose Metabolism and Pentose Phosphate Pathway (PPP) Activation During Liver Regeneration

Figure S1 revealed that PHx triggered nucleotide catabolism, with accumulation of purine intermediates IMP and inosine, reflecting energy stress and rapid nucleotide breakdown. Conversely, RRP treatment activated both pyrimidine and purine synthesis pathways, increasing intermediates such as UMP and GMP, thus providing essential nucleic acid precursors for hepatocyte proliferation. The RRP-related specific rise in citicoline (CDP-choline) further supported membrane phospholipid synthesis, promoting cell membrane repair and regeneration. Figure S2 highlighted a pivotal role of PPP in liver regeneration. The PHx group showed increased levels of D-glucose 6-phosphate and 6-phosphogluconic acid, indicating activation of the oxidative branch of the PPP, crucial for NADPH production and antioxidative defense. In the RRP group, enhanced accumulation of D-ribulose 5′-phosphate from the non-oxidative PPP branch further supported the biosynthetic demand for nucleotides. Additionally, elevated S-lactoylglutathione levels reflected strengthened glutathione conjugation metabolism, collectively boosting the antioxidant capacity of the liver. Figure S3 emphasized the potential role of glucogenic amino acids in liver regeneration. L-threonine was significantly upregulated in the RRP group, serving as a key glucogenic amino acid that replenishes glucose intermediates to meet energy demands. RRP also modulated intrahepatic levels of L-asparagine and L-ornithine, which are involved in glucogenic pathways and nitrogen metabolism, balancing glucose production and reducing detoxification burden, thereby optimizing metabolic flexibility for regenerating hepatocytes.

**Figure 7:**
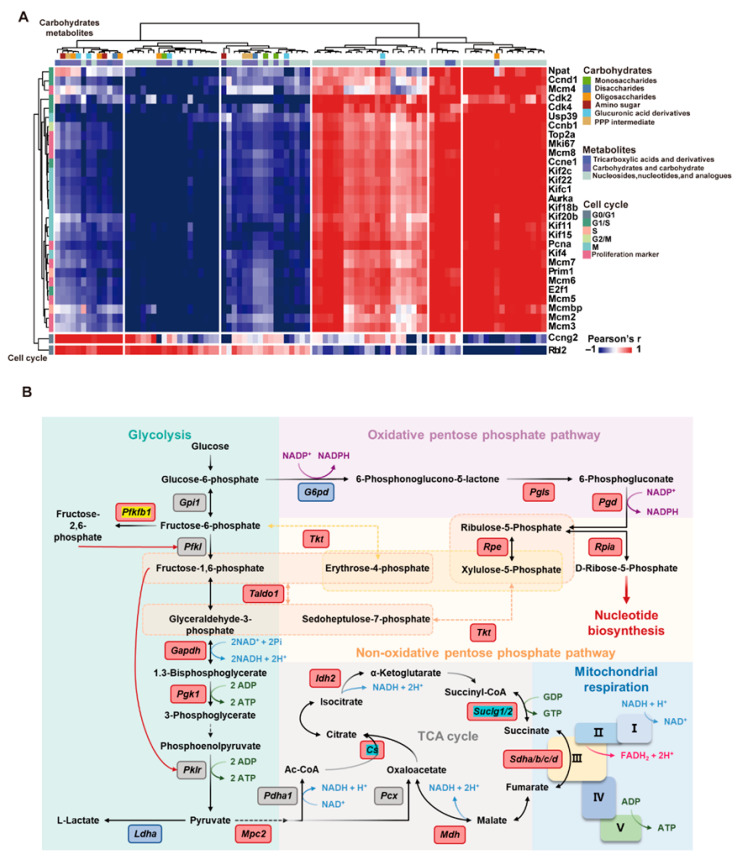
RRP coordinates carbohydrate metabolism with cell cycle progression to support liver regeneration. (**A**) Correlation heatmap of carbohydrate metabolites and cell cycle genes. (**B**) Schematic map of metabolic pathways with highlighted upregulated genes.

Figure 7A illustrated the global association patterns between cell cycle-related genes and carbohydrate metabolites, as revealed by Pearson correlation analysis. Approximately 60% of gene–metabolite pairs showed significant positive correlations, 25% showed significant negative correlations, and the remaining 15% exhibited no clear association. Specifically, the core proliferation genes expression (Pcna, Ki67) displayed strong positive correlations with nucleotide biosynthesis-related metabolites (such as PPP intermediates and nucleotide analogs), underscoring the metabolic demands of DNA replication. The expression levels of G1 phase initiator genes (Ccnd1, Cdk4) were negatively correlated with mono- and disaccharides, implying the massive consumption of carbohydrates during the cell cycle initiation. TCA cycle-derived metabolites showed moderate positive correlations with the expression of mitosis-related genes (Cdk1, Top2a), reflecting the energy requirement of cell division. Notably, several carbohydrate metabolites, such as glucosamine, were positively correlated with the expression of motor proteins, like Kinesin Family Member 18B (Kif18b), suggesting a possible connection between cytoskeletal regulation and hepatocyte proliferation. Together, these findings highlighted a tightly coordinated and dynamic interplay between cell cycle progression and carbohydrate metabolism.

Transcriptomic analysis further corroborated these metabolomic findings. RRP promoted glycolysis, suppressed gluconeogenesis, and enhanced PPP-related gene expression (G6pd, Pgd), thereby promoting NADPH generation for antioxidation and nucleotide synthesis. Furthermore, RRP restored gene expression associated with TCA cycle and mitochondrial function (Cs, Aco1, Sdha) and downregulated Pdk1 to relieve inhibition on pyruvate dehydrogenase to facilitate the entry of pyruvate into the TCA cycle. RRP also upregulated Phgdh to support serine biosynthesis, one-carbon metabolism, and methyl donor generation (Figure 7A and Figure S4). These findings indicate that RRP enhanced mitochondrial energy production while simultaneously promoting NADPH generation and nucleotide biosynthesis via the PPP, orchestrating comprehensive metabolic reprogramming that supports hepatocyte proliferation (Figure 7B).

### 3.6. RRP Protects Mitochondria During Early Liver Regeneration

**Figure 8:**
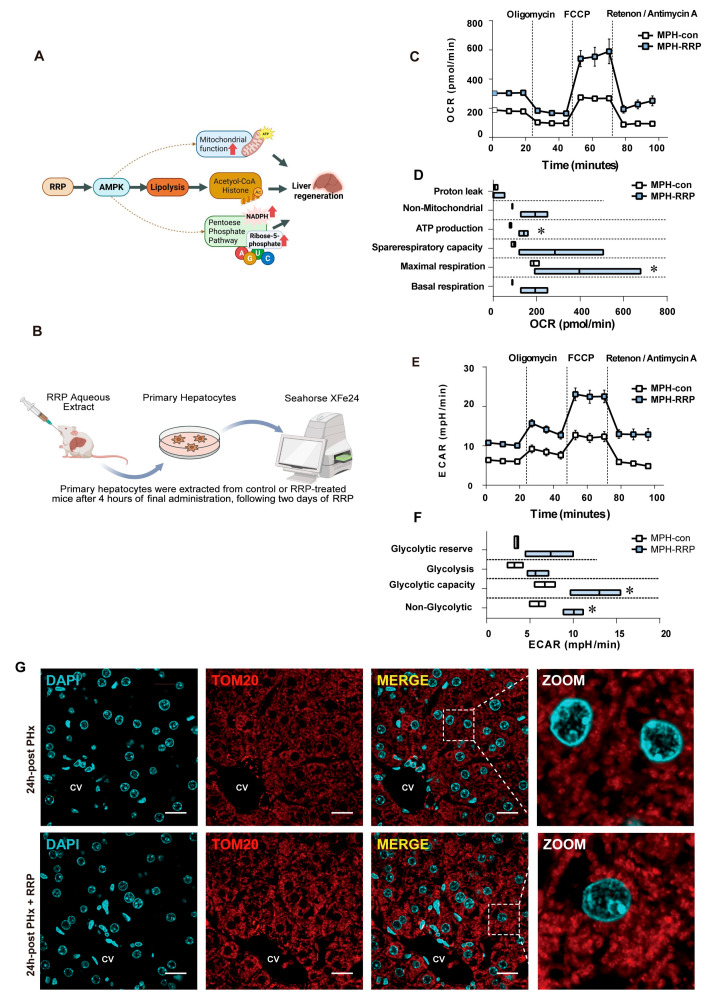
RRP improves mitochondrial respiration and glycolysis to meet energy demand during liver regeneration. (**A**) Workflow of Seahorse XFe24 analysis in primary hepatocytes. (**B**,**C**) OCR profiles and quantification of mitochondrial respiration in primary hepatocytes. (**D**,**E**) ECAR profiles and quantification of glycolytic function. (**F**) Immunofluorescence staining of TOM20 and DAPI in liver sections at 24 h post PHx with or without RRP treatment. Scale bar = 20 μm. (CV represents the central vein). (**G**) Diagram illustrating the mechanism by which RRP promotes liver regeneration. Statistical significance: * *p* < 0.05 as compared with the control group.

Since the lipid and glucose metabolic processes described above depended on mitochondrial functional integrity, we finally examined how RRP affected mitochondrial function itself during early liver regeneration. Figure 8A illustrates the overall mechanism of RRP treatment and its effects on liver regeneration through mitochondrial and metabolic pathways. To capture the in vivo effects, mice were pretreated with RRP by oral gavage once daily for two consecutive days. Four hours after the final administration, primary hepatocytes were isolated from control and RRP-treated mice for immediate metabolic analysis using the Seahorse XFe24 extracellular flux analyzer (Figure 8B). Oxygen consumption rate (OCR) measurements revealed that hepatocytes from RRP-treated mice showed significantly increased ATP production and maximal respiratory capacity, indicating enhanced mitochondrial oxidative phosphorylation. Other OCR parameters, such as proton leak, basal respiration, non-mitochondrial respiration, and spare respiratory capacity, remained unchanged, suggesting that mitochondrial integrity was preserved (Figure 8C,D). Concurrently, extracellular acidification rate (ECAR) analysis demonstrated elevated basal and compensatory glycolysis, reflecting increased glycolytic capacity (Figure 8E,F). Immunofluorescence staining for TOM20, a key component of the mitochondrial outer membrane translocase complex responsible for importing nuclear-encoded proteins, showed increased expression and more distinct mitochondrial morphology in RRP-treated mouse livers (Figure 8G). These results indicated that RRP treatment improved mitochondrial biogenesis and metabolic efficiency in hepatocytes to support the energy demands of liver regeneration.

## 4. Discussion

Research on liver regeneration has laid a theoretical foundation for treating acute liver injury, improving recovery after partial hepatectomy, and managing end-stage liver diseases [29]. Enhancing regeneration efficiency is critical for reducing the risk of postoperative liver failure, which remains a major clinical challenge. In this study, we demonstrated that RRP significantly promoted liver regeneration during both the early and acceleration phases following partial hepatectomy. Mechanistically, RRP activated AMPK signaling pathway, inducing metabolic reprogramming that prioritized energy production and facilitated cell cycle progression. The inhibition of AMPK disrupted this coordination, leading to impaired regeneration, thereby highlighting AMPK as a key mediator of the pro-regenerative effects of RRP.

These findings can be corroborated by previous studies, which have demonstrated that the activation of AMPK is associated with the therapeutic effects of RRP in other pathological conditions [25,30]. Luo et al. had also shown that RRP alleviated hepatic ischemia–reperfusion injury via AMPK-mediated inhibition of SREBP2-driven cholesterol synthesis and the activation of LXRα-dependent cholesterol efflux [22]. Among various active components in RRP, verbascoside was reported to mitigate hepatocyte damage by downregulating NR4A1 expression, thereby reactivating the LKB1-AMPK pathway [31]. Catalpol and verbascoside, have also been shown to robustly activate AMPK [32,33]. In our study, Western blot analysis confirmed that RRP significantly activated AMPK signaling, as evidenced by increased phosphorylation of AMPK and the degradation of its downstream target ACC. This shift promoted FAO, reduced lipid accumulation, and elevated hepatic acetyl-CoA levels, a critical metabolite fueling the TCA cycle and serving as a substrate for histone acetylation. Similar regulatory convergence between metabolic reprogramming and epigenetic modulation has also been observed in hepatocellular carcinoma, where fatty acid metabolic plasticity contributes to tumor heterogeneity and reveals potential therapeutic targets [34]. In our previous work, we demonstrated that increased acetyl-CoA enhances H3K27ac modifications and activates cell cycle genes such as Ccnd1 [10]. Consistently, RRP treatment upregulated both acetyl-CoA levels and H3K27ac marks in regenerating liver tissue. These findings provide direct evidence that the activation of AMPK mediated by RRP promotes hepatocyte regeneration driven by metabolic reprogramming. However, the specific active compounds within RRP that are responsible for these effects, as well as their pharmacokinetic profiles, have not yet been fully characterized [35,36]. Given that various polysaccharides from Chinese herbal medicine have shown protective effects against liver injury via metabolic regulatory pathways [37], it is plausible that the heteropolysaccharides from RRP, which are rich in galactose and glucose, may also promote liver regeneration [38]. Additionally, RRP contains glycosides with hepatoprotective properties. For instance, acteoside can alleviate hepatocyte ferroptosis and ischemia–reperfusion injury by targeting PCBP2 [39], while catalpol enhances insulin sensitivity and mitochondrial respiration through the activation of the AMPK/SIRT1/PGC-1α/PPAR-γ pathway [40]. However, whether these candidate active ingredients can truly represent the liver-regenerating effects of RRP requires further research for validation. This gap in knowledge limits our mechanistic understanding and hampers the translation of these findings into effective drug discovery strategies.

In murine models, precise regulation of glycolysis and oxidative phosphorylation was essential for effective liver regeneration as well. Excessive glycolysis, as induced by PP2Acα deletion, delayed the termination of regeneration, and enhanced glycolytic flux resulting from PDK4 deficiency accelerated hepatocyte proliferation through improved insulin signaling and activation of the AMPK/FOXO1/CD36 axis [41]. Interestingly, impairment of mitochondrial oxidative phosphorylation, as observed in FXR activation models, also hindered liver regeneration and highlighted the importance of mitochondrial ATP production [42]. RRP appeared to coordinate glycolysis and mitochondrial function in a way that aligned metabolic output with regenerative demand. Rather than triggering isolated metabolic shifts, RRP induced a broader adjustment in energy metabolism by promoting pyruvate entry into the TCA cycle, improving mitochondrial oxidative efficiency, and supporting acetyl-CoA accumulation, thereby establishing a reprogrammed metabolic state optimized for hepatocyte proliferation. This pattern echoed regenerative models such as PDK4 deficiency, where improved metabolic coupling accelerated liver regrowth. Together, these findings indicated that RRP promoted liver regeneration by reinforcing an integrated and metabolically adaptable cellular state.

In addition to its role in energy metabolism, RRP significantly influenced nucleotide biosynthesis, a process essential for cell proliferation and tissue repair [43]. The critical role of de novo nucleotide synthesis in liver regeneration has been validated in various models [44]. Pharmacological inhibition of pyrimidine synthesis using agents like methotrexate [45], 5-Fluorouracil [46], or dihydroorotate dehydrogenase inhibitors [47] severely impaired hepatocyte proliferation after PHx, and could only be rescued by exogenous nucleoside supplementation. Similarly, disruption of the PPP significantly diminished the regenerative capacity of hepatocytes. Deletion of key regulators such as NRF2, BMAL1, and HIF-1α also led to reduced expression of G6PD, the rate-limiting enzyme of the PPP [48,49]. This reduction in turn caused insufficient NADPH production, which is crucial for maintaining redox balance and supporting nucleotide biosynthesis during hepatocyte proliferation. The resulting nucleotide deficiency impaired DNA replication, thereby hindering the regenerative process. However, restoring PPP activity in these compromised settings partially rescued hepatocyte regeneration, thus underscoring the critical importance of the PPP in supporting liver regenerative capacity [50]. In our study, untargeted metabolomics revealed increased levels of intermediates involved in nucleic acid synthesis after RRP treatment, indicating enhanced precursor availability for DNA and RNA production. Enrichment analysis further showed upregulation of PPP-related genes such as Tkt, Taldo1, Rpe, and Ppia. By modulating G6P flux and regulating PPP-related gene expression through downstream targets like FOXO1 and NRF2, RRP-mediated activation of AMPK coordinated biosynthetic and antioxidant demands. These findings suggest that RRP promotes PPP activity and nucleotide synthesis, thereby supporting the metabolic needs of regenerating liver tissue.

Our study integrated metabolic phenotyping with mechanistic investigation to demonstrate the therapeutic potential of RRP on liver regeneration. By linking acetyl-CoA flux, fatty acid oxidation, and PPP activation to proliferative signaling, it proposes a coordinated and targetable metabolic framework for enhancing regeneration. There are still limitations to be addressed in the future. Firstly, upstream metabolic sensors and downstream chromatin/transcriptional regulators were not fully explored, and the active constituents of RRP have yet to be identified. Furthermore, the effects of RRP on other tissues related to energy and metabolism, such as muscles and adipocytes, have not been investigated. These tissues, particularly adipose tissue and skeletal muscle, may contribute to liver regeneration through mechanisms such as fatty acid mobilization, lactate cycling, and amino acid supply [51,52]. Future studies should address these gaps to fully understand the systemic metabolic impact of RRP.

## 5. Conclusions

In conclusion, our study demonstrated that RRP promoted liver regeneration after PHx by activating AMPK signaling. AMPK activation coordinated various metabolic pathways, including lipolysis with increased acetyl-CoA supplement, enhanced mitochondrial function, and redirected glucose metabolism into the pentose phosphate pathway, thereby supporting hepatocyte proliferation and tissue repair. These findings elucidate the pivotal role of AMPK signaling in metabolic regulation during liver regeneration, and underscore the therapeutic potential of RRP in mitigating morbidity and mortality associated with impaired regenerative capacity.

## Data Availability

The data that support the findings of this study are available on request from the corresponding author upon reasonable request.

## Supplementary Materials

The following supporting information can be downloaded at: https://www.mdpi.com/article/10.3390/nu17223579/s1. Figure S1: Heatmap of nucleotide-related metabolites in liver tissues from PHx and RRP-treated mice at 24 h post-surgery. Figure S2: Heatmap of carbohydrate-related metabolites in liver tissues from PHx and RRP-treated mice at 24 h post-surgery. Figure S3: Heatmap showing changes in amino acid and peptide metabolites in liver tissues from PHx and RRP-treated mice at 24 h post-surgery. Figure S4: Heatmap of representative genes involved in glycolysis, gluconeogenesis, TCA cycle, oxidative phosphorylation, and pentose phosphate pathway in liver tissues from the control, PHx, and RRP groups.

## Abbreviations

RRP, Radix Rehmanniae Praeparata; PHx, partial hepatectomy; AMPK, AMP-activated protein kinase; ACC, Acetyl-CoA carboxylase; p-ACC, phosphorylated ACC; p-AMPK, phosphorylated AMPK; ALT, alanine aminotransferase; AST, aspartate aminotransferase; Ccnd1, cyclin D1; Ki67, Marker of Proliferation Ki-67; H3K27ac, Histone H3 lysine 27 acetylation; CC, Compound C; DEGs, differentially expressed genes; ECAR, extracellular acidification rate; FAO, fatty acid oxidation; FXR, farnesoid X receptor; GO, Gene Ontology; G6PD, glucose-6-phosphate dehydrogenase; KEGG, Kyoto Encyclopedia of Genes and Genomes; NADPH, nicotinamide adenine dinucleotide phosphate; OAPA, oleic acid/palmitic acid mixture; OCR, oxygen consumption rate; PPP, pentose phosphate pathway; PCA, principal component analysis; qPCR, quantitative real-time polymerase chain reaction; ROS, reactive oxygen species; R5P, ribose-5-phosphate; SEM, standard error of the mean; SREBP1, sterol regulatory element-binding protein 1; TCM, Traditional Chinese Medicine; TCA, tricarboxylic acid cycle.
