# Supplementary material for: Radix Rehmanniae Praeparata Extract Enhances Liver Regeneration Through AMPK-Driven Metabolic Reprogramming
Source: Nutrients. 2025 Nov 15;17(22):3579. doi: 10.3390/nu17223579 (PMC12655178; doi:10.3390/nu17223579)
Supplement: Supplementary file 1 [file nutrients-17-03579-s001.zip › nutrients-3948539-supplementary.pdf]

Supplementary files

Supplementary figure legends

Supplementary Figure 1

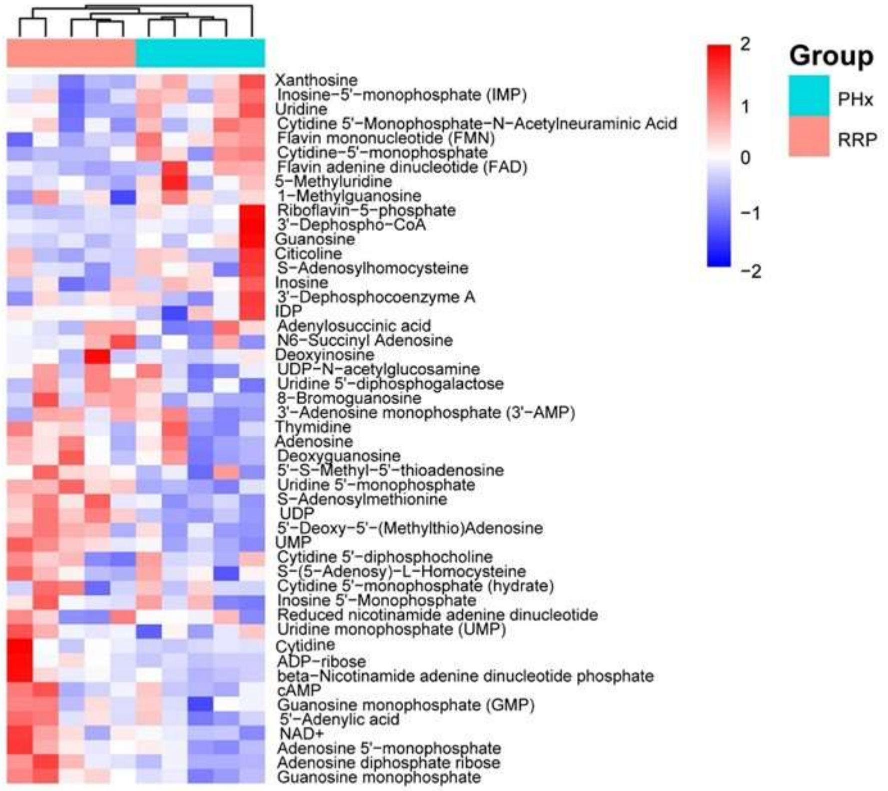

Supplementary Figure S1. Heatmap of nucleotide-related metabolites in liver tissues from PHx and RRP-treated mice at 24 h post-surgery.

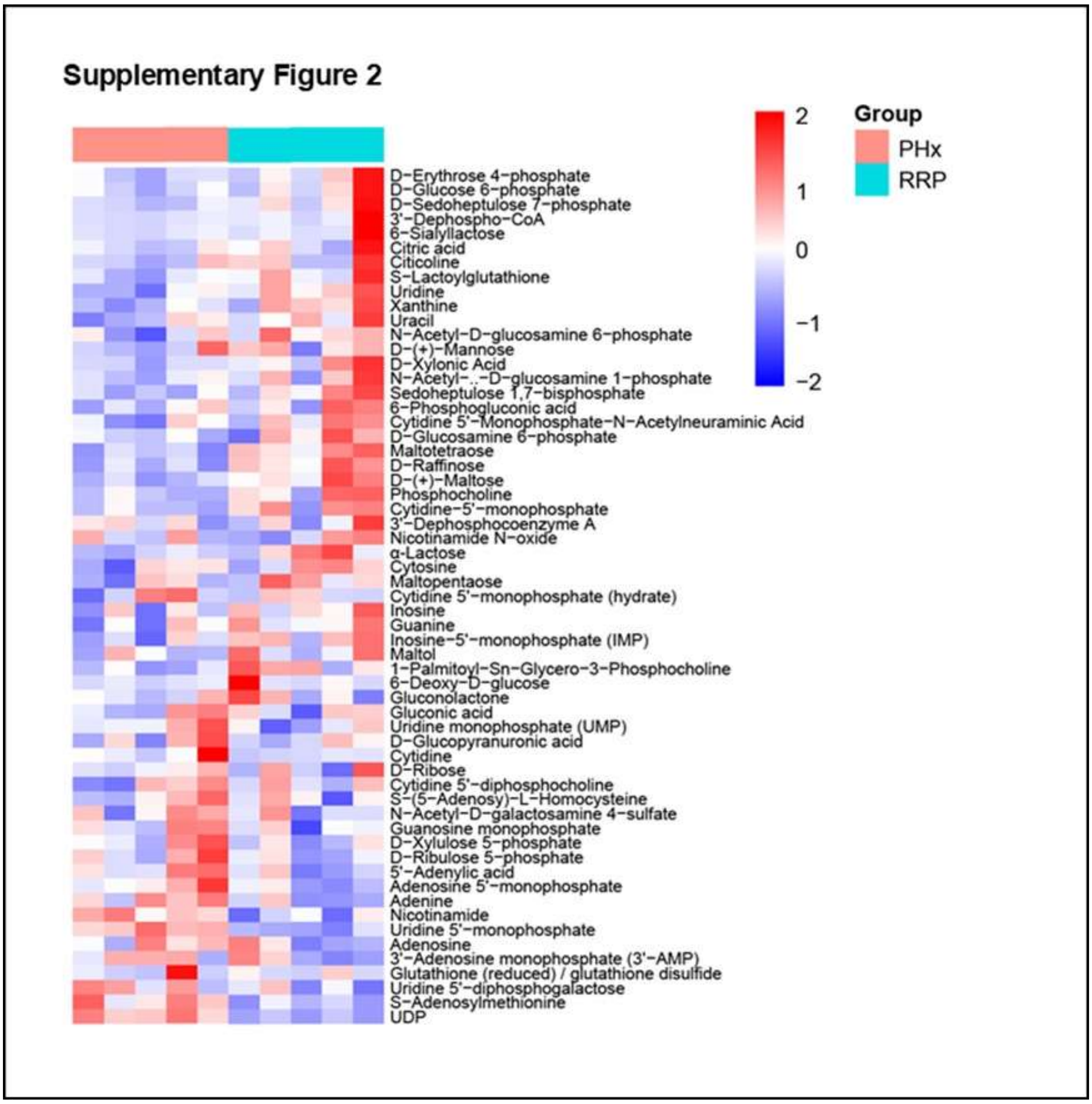

Supplementary Figure S2. Heatmap of carbohydrate-related metabolites in liver tissues from PHx and RRP-treated mice at 24 h post-surgery.

Supplementary Figure 3

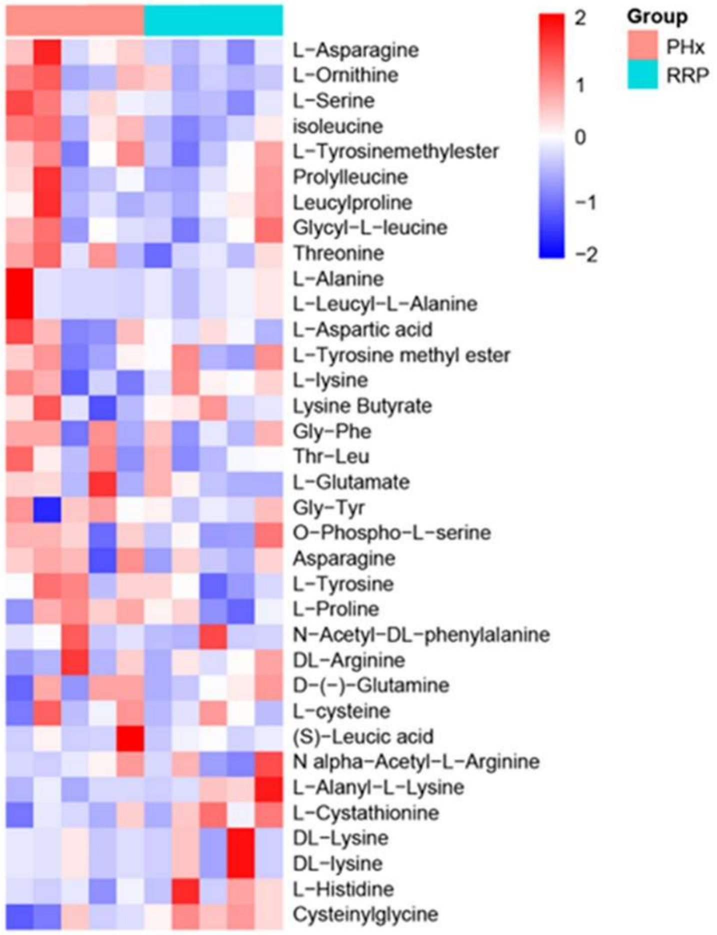

Supplementary Figure S3. Heatmap showing changes in amino acid and peptide metabolites in liver tissues from PHx and RRP-treated mice at 24 h post-surgery.

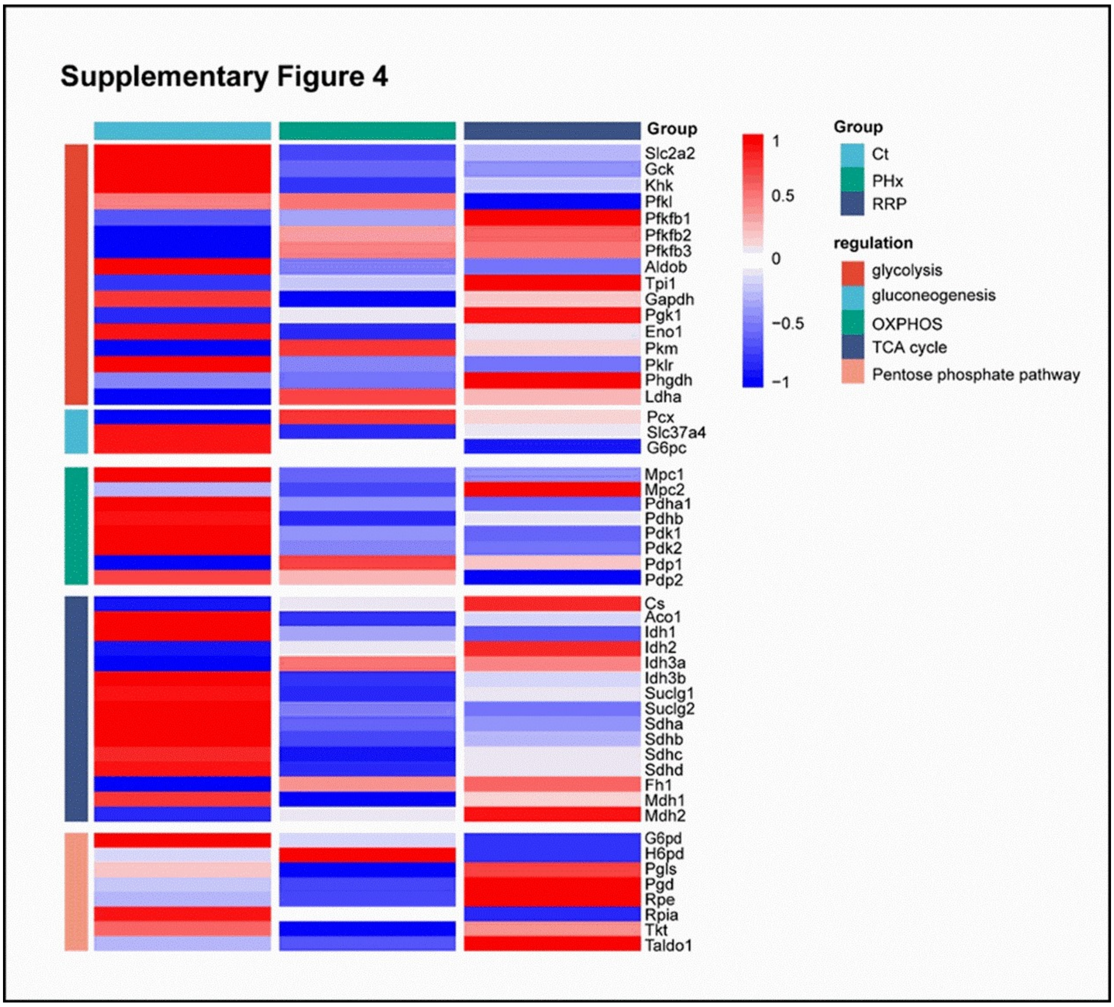

Supplementary Figure S4. Heatmap of representative genes involved in glycolysis, gluconeogenesis, TCA cycle, oxidative phosphorylation, and pentose phosphate pathway in liver tissues from the control, PHx, and RRP groups.
